# Prefrontal Dopaminergic and Enkephalinergic Synaptic Accommodation in HIV-associated Neurocognitive Disorders and Encephalitis

**DOI:** 10.1007/s11481-012-9345-4

**Published:** 2012-03-06

**Authors:** Benjamin B. Gelman, Joshua G. Lisinicchia, Tianshen Chen, Kenneth M. Johnson, Kristofer Jennings, Daniel H. Freeman, Vicki M. Soukup

**Affiliations:** 1 Department of Pathology, University of Texas Medical Branch, 301 University Blvd., Galveston, TX 77555-0609 USA; 2 Department of Preventive Medicine & Community Health, University of Texas Medical Branch, 301 University Blvd., Galveston, TX 77555-0609 USA; 3 Department of Pharmacology, University of Texas Medical Branch, 301 University Blvd., Galveston, TX 77555-0609 USA; 4 Department of Neurology, University of Texas Medical Branch, 301 University Blvd., Galveston, TX 77555-0609 USA; 5 University of Texas Medical Branch, Keiller Building, 3.118, Route 0609, Galveston, TX 77555-0609 USA

**Keywords:** Dopamine receptor, Enkephalin, HAND, HIVE, HIV encephalitis, HIV-associated neurocognitive disorders, Interferon regulatory factor, Opiate, Opioid, Synaptic plasticity

## Abstract

Changes in synapse structure occur in frontal neocortex with HIV encephalitis (HIVE) and may contribute to HIV-associated neurocognitive disorders (HAND). A postmortem survey was conducted to determine if mRNAs involved in synaptic transmission are perturbed in dorsolateral prefrontal cortex (DLPFC) in subjects with HIVE or HAND. Expression of the opioid neurotransmitter preproenkephalin mRNA (*PENK*) was significantly decreased in a sampling of 446 brain specimens from HIV-1 infected people compared to 67 HIV negative subjects. Decreased DLPFC *PENK* was most evident in subjects with HIVE and/or increased expression of interferon regulatory factor 1 mRNA (*IRF1*). Type 2 dopamine receptor mRNA (*DRD2L*) was decreased significantly, but not in the same set of subjects with *PENK* dysregulation. *DRD2L* downregulation occurred primarily in the subjects without HIVE or neurocognitive impairment. Subjects with neurocognitive impairment often failed to significantly downregulate *DRD2L* and had abnormally high *IRF1* expression. Conclusion: Dysregulation of synaptic preproenkephalin and *DRD2L* in frontal neocortex can occur with and without neurocognitive impairment in HIV-infected people. Downregulation of *DRD2L* in the prefrontal cortex was associated with more favorable neuropsychological and neuropathological outcomes; the failure to downregulate *DRD2L* was significantly less favorable. *PENK* downregulation was related neuropathologically to HIVE, but was not related to neuropsychological outcome independently. Emulating endogenous synaptic plasticity pharmacodynamically could enhance synaptic accommodation and improve neuropsychological and neuropathological outcomes in HIV/AIDS.

## Introduction

HIV-1-associated neurocognitive disorders (HAND) remain highly prevalent in the era of highly active antiretroviral therapy (HAART) (Heaton et al. 2010; McArthur et al. 2010). Without HAART the pathophysiology of HAND was significantly linked with high replication rates of HIV-1 in the central nervous system (CNS) and a CNS inflammatory reaction known as HIV encephalitis (HIVE) (Budka et al. 1991). In the era of HAART about 50% of subjects have neurocognitive impairment to some extent (Heaton et al. 2010) but less than 10% have a neuropathological diagnosis of HIVE (Masliah et al. 2000; Everall et al. 2009). Early in the pandemic it was recognized that HIVE was not present in many subjects with neurocognitive impairment (Glass et al. 1995; Wiley and Achim 1994), and the divergence may have widened in the HAART era (Everall et al. 2009; Gelman 2007). An important role for altered synaptic transmission in HIVE has been widely suggested in the literature. Synaptodendritic simplification is a neurodegenerative type of change that occurs in HAND with HIVE (Masliah et al. 1997), and forms a potential therapeutic concept for translational neuroscience (Gelbard et al. 2010). Transcriptional regulation of genes that code for presynaptic proteins and postsynaptic neurotransmitter receptors and ion channels are perturbed in the dorsolateral prefrontal cortex (DLPFC) of people with HIVE (Masliah et al. 2004; Gelman et al. 2004). Isolated synapses from DLPFC in HIVE contain proteomic changes and altered protein management (Gelman and Nguyen 2010; Nguyen et al. 2010). Many clinical and experimental observations suggest that specific neurotransmitter systems and circuits are disturbed. Examples include striatal dopaminergic circuits (Hriso et al. 1991; Berger and Arendt 2000; Nath et al. 2000; Nath et al. 2002; Wang et al. 2004a, b; Gelman et al. 2006), basal forebrain cholinergic circuits (Sarter and Podell 2000; Koutsilieri et al. 2000, 2001a), peptidergic systems including tachykinins, somatostatin and glutamate (Koutsilieri et al. 2001b; Ho and Douglas 2004; Da Cunha et al. 1995; Ernst et al. 2010; Ferrarese et al. 2001; Meisner et al. 2008; Kovacs and De Wied 1994; Koutsilieri et al. 2001b), and serotonergic systems (Schroecksnadel et al. 2007; Murray 2003). Collectively, anomalies in synaptic transmission imply that neuronal excitability and circuit functions may be abnormal in HAND. In turn, neuroprotective strategies that focus on neuronal excitation and synaptic plasticity could stabilize neural circuitry and prevent HAND (Gelman et al. 2004; Di Filippo et al. 2008; Gelbard et al. 2010). Prefrontal dopaminergic circuits in HIV/AIDS are especially important because they undergo disease-associated changes in synaptic tone that produce abnormal neurocognitive phenotypes (Goldman-Rakic 1996; Goldman-Rakic 1998; El-Ghundi et al. 2007; Kroener et al. 2009). Those phenotypes strongly resemble many of the behavioral anomalies that occur in HAND (Chang et al. 2008, Heaton et al. 2010; Woods et al. 2009).

In patients with HAND and HIVE the putative driving force is a combination of brain inflammatory mediators and HIV-1 proteins that participate in multicellular networks that damage “bystander” neuronal elements including synapses (Kaul and Lipton 2006). Many inflammatory mediators expressed in HIVE can produce functional changes in synaptic transmission experimentally. Interferons (IFNs), cytokines including tumor necrosis factor alpha and interleukins influence synaptic long term potentiation (D’Arcangelo et al. 1991; Jankowsky and Patterson 1999; Tancredi et al. 1992; Dafny et al. 1996; Mendoza-Fernandez et al. 2000; Cunningham et al. 1996; Ross et al. 2003; Hadjilambreva et al. 2005; Dafny and Yang 2005; Maher et al. 2006; Khairova et al. 2009), synaptic plasticity (Albensi and Mattson, 2000; Boulanger et al. 2001; Boulanger 2004; Stellwagen and Malenka 2006; Jakubs et al. 2008; Kawasaki et al. 2008; Steinmetz and Turrigian 2010; Yirmiya and Goshen 2011), and synaptogenesis and maintenance (O’Connor and Coogan 1999; Dafny et al. 1996; Vikman et al. 2001; Kim et al. 2002; Brask et al. 2004; Mizuno et al. 2008; Boulanger 2009; Victorio et al. 2010). IFNs are especially important in HIVE because interferon responsive genes (IFRGs) are the most regulated brain gene expression pathway observed on arrays (Masliah et al. 2004). HIVE is likely to be a prototypal clinical example of neuroimmune-associated synaptic dysfunction, but those interrelationships have not been well-characterized in HIV-1-infected brain specimens. To address this we performed neurochemical studies in 513 human brain specimens spanning over two decades of the HIV/AIDS pandemic. Dorsolateral prefrontal cortex (DLPFC) was highlighted because the functional output of DLPFC is abnormal in HAND (Heaton et al. 2010; Woods et al. 2009). Two abnormal neurotransmitter systems were identified and characterized. Associations are addressed concerning frontal lobe dysfunction, neuroimmune changes, neurovirological status, neuropathology and key comorbidities.

## Methods

### Brain specimens

Preproenkephalin mRNA (*PENK*) and type 2 dopamine receptor long splice isoform (*DRD2L*) mRNA concentrations were assayed in a total of 513 frozen human brain samples. 446 were HIV infected patients and 67 were age- gender- and ethnicity-comparable HIV seronegative decedents who died during a comparable time span (Table 1) (Nguyen et al. 2010). 312 out of 446 HIV-infected subjects died in the HAART era (1998 to 2011) and were obtained from the National NeuroAIDS Tissue Consortium (NNTC) (Morgello et al. 2001). Written consent was obtained for subjects at four collection sites in the USA. The following offices maintained institutional review boards (IRBs) that provided oversight for the protection of human subjects: 1) The University of Texas Medical Branch Office of Research Subject Protections; 2) Mount Sinai Medical Center Program for the Protection of Human Subjects; 3) University of California, San Diego Human Research Protections Program; 4) University of California, Los Angeles Office of the Human Research Protection Program. These IRBs reviewed the protocol at regular intervals and gave written approvals continuously over 12 consecutive years to the present. Most HAART-era subjects underwent neurocognitive evaluations and substance use surveys using the NNTC protocol, as indicated in tables and figures. 134 of the decedents with HIV/AIDS were archived in the 20^th^ Century and prior to HAART (between 1988 and 1996) in Galveston, Texas, USA. Texan subjects who underwent a complete autopsy before the NNTC did not undergo a structured neurocognitive evaluation; their pathological characteristics have been reported (Gelman et al. 1996).

**Table 1:** HIV positive decedents with and without HIV encephalitis and seronegative controls

Characteristic	HIV Infection^a^		p	HIV encephalitis^b^		p
	HIV-	HIV+	HIV- vs HIV+	HIVE-	HIVE+	HIVE- vs HIVE+
Number of subjects	67	446	N/A	354	92	N/A
Age^c^	46.9 ± 13.6	42.6 ± 9.6	0.001	43 ± 10	41.2 ± 7.8	0.106
Gender (M/F)^d,e^	53/14	384/62	0.133	299/55	85/7	0.050
Race (W/B/A/O)^e,f^	41/20/0/6	271/146/6/23	0.468	218/113/6/17	53/33/0/0	0.091
Hispanic or Latino (yes/no)^e^	16/51	88/358	0.431	65/289	23/69	0.154
Postmortem freezing interval^c^	15 ± 11.5	14.5 ± 14.7	0.787	14.1 ± 13.4	16.0 ± 19	0.268
Log_10_ Plasma HIV RNA (copies/ml)^c^ (*n* = 263)	N/A^g^	4.2 ± 1.5	N/A	4.0 ± 1.6	5.1 ± 0.9	<0.001
Log_10_ Cerebrospinal fluid HIV RNA (copies/ml)^c^ (*n* = 191)	N/A	2.8 ± 1.5	N/A	2.6 ± 1.3	4.2 ± 1.7	<0.001
Blood CD4+ lymphocyte count (cells/mm^3^)^c^ (*n* = 272)	N/A	106 ± 162	N/A	119 ± 175	52.3 ± 64.5	0.007

^a^HIV-, Human Immunodeficiency Virus seronegative; HIV+, seropositive

^b^HIVE, HIV encephalitis

^c^Mean ± standard deviation, Student’s *t* test

^d^M/F, Male/female

^e^Chi square

^f^B/W/A/O, Black/White/Asian/Other or not known

^g^N/A, not applicable

### Neurocognitive testing, substance use survey, neuropathology

Two hundred seventy two out of 312 HAART-era subjects were clinically assessed for neurocognitive impairment by a neuropsychologist. 218 out of 272 underwent a structured battery of neurocognitive tests employed by the NNTC at 6 month intervals until autopsy (Morgello et al. 2001). All results were obtained within 6 months prior to death. A composite normalized impairment T score was obtained, and seven normalized component neurocognitive domain T scores were evaluated. The consistency of neurocognitive testing across the four NNTC specimen collection sites was maintained with a structured quality assurance program. Evaluations were scored according to age-adjusted norms and clinical diagnoses were assigned according to modified American Academy of Neurology criteria (Woods et al. 2004). One of four neurocognitive diagnoses was considered: a) No syndromic neurocognitive impairment; b) mild cognitive and motor disorder (MCMD); c) HIV-1-associated dementia (HAD); d) impairment possibly caused by medical factors other than HIV/AIDS (NPI-O). A diagnosis of syndromic impairment included the diagnoses of MCMD and HAD, but not NPI-O. The nosological diagnosis of HAND (Antinori et al. 2007) was not used in the present study because: 1) very few subjects in the NNTC autopsy cohort have a diagnosis of “no HAND,” and 2) this study and the NNTC began before the establishment of the nosological diagnosis of HAND.

The Psychiatric Research Interview for Substance and Mental Disorders (PRISM) was used to obtain self-reported lifetime histories of substance abuse and dependence. A quality assurance program was followed to maintain consistency across the four NNTC test sites (Morgello et al. 2006). Neuropathological diagnoses were rendered by NNTC site neuropathologists (B. Gelman, Texas; S. Morgello, New York; E. Masliah, San Diego; D. Commons, Los Angeles). Consistency across all four NNTC collection sites was maintained by using a uniform sampling strategy and following a structured program of quality assurance that consisted of frequent face-to-face reviews using a multi-head microscope. Criteria used for the nosological diagnosis of HIVE were according to Budka et al. (1991).

### Brain dissection and RNA isolation

DLPFC was emphasized because gene arrays and proteomic studies indicate that synaptic disturbances occur in that sector (Masliah et al. 2004; Gelman and Nguyen 2010). Frozen samples of DLPFC were dissected from Brodmann areas 9 or 10. The Qiagen RNeasy Lipid Tissue Mini Kit (Cat. No. 74804, Valencia, CA, USA) was used to prepare RNA. About 100 mg of brain tissue was dissected on dry ice and placed in 1 ml of QIAzol and 0.5 g of 0.5 mm Zirconia/Silica beads (Cat. 11079105z, BioSpec Products, Bartlesville, OK, USA). Samples were homogenized using three 20 s pulses in a mini-bead beater. At room temperature 200 μl chloroform was added with shaking for 15 s, and after standing 3 min was centrifuge at 12,000 x g for 15 min at 4°C. One volume of 70% ethanol was added to the aqueous phase with mixing. Up to 700 μl of the extract was added to RNeasy mini spin columns in a volume of 2.0 ml. After centrifuging for 15 s at 8000 x g at room temperature the spin was repeated with the remainder of the extract. Columns were washed with 350 μl Buffer RW1 and treated with RNase-free DNase I (Cat. 79254, Qiagen, Valencia, CA, USA) at room temperature for 15 min. 350 μl Buffer RW1 was added to the columns and centrifuged for 15 s at 8000 x g. Columns were washed with 500 μl Buffer RPE twice, and re-centrifuged at maximum speed for 2 min to remove salts. RNA was eluted twice with 50 μl RNase-free water.

### Assay of PENK, DRD2L, IRF1, GAPDH and HIV-1 gag/pol, mRNA expression

Bio-Rad iScript cDNA Synthesis Kit (Cat. No. 170-8891, Hercules, CA, USA) was used to synthesize cDNA. 1 μg of brain RNA, 4 μl of 5x iScript reaction mix, 1 μl of iScript reverse transcriptase was adjusted to 20 μl with nuclease-free water. The mixture was incubated for 5 min at 25°C, 30 min at 42°C, 5 min at 85°C, then held at 4°C. HIV cDNA was prepared using HIV anti-sense primer 84R in a final concentration of 1 μmol/L (Palmer et al. 2003). *PENK* primer sequences used were: AGAAGGCGAAAGTTACTCCAA and CACCATCAACAGTTTCCCAC; *PENK* probe sequence: Fam-ACTAGTGGCCCCAGGCCCCA-TAMRA. Glyceraldehyde 3-phosphate dehydrogenase (*GAPDH*) was used as normalizing gene. *GAPDH* primer sequences: GAAGGTGAAGGTCGGAGTC and GAAGATGGTGATGGGATTTC; *GAPDH* probe sequence: Fam-CAAGCTTCCCGTTCTCAGCC-TAMRA. All reagents were from Sigma Genosys (The Woodlands, TX, USA). For each reaction, 12.5 μl of 2x JumpStart Taq ReadyMix for Quantitative real time PCR (Sigma, St. Louis, MO, USA) (Cat. No. D7440) was combined with 1 μl of cDNA, 3.5 μl of 25 mmol/l MgCl_2_, 0.8 μl of 10 μmol/l primer mix and 0.5 μl of 10 μmol/l probe adjusted to a volume to 25 μl with water. PCR conditions were 2 min at 95°C, 40 cycles of 15 s at 95°C and 60 s at 60°C. For assay of the long splice isoform of the type 2 dopamine receptor, 1 μl of 20x *DRD2L* primers and probe mix (Cat. Hs01024460_m1, Applied Biosystems, Foster City, CA, USA) was combined with 1 μl of cDNA, 10 μl of 2x JumpStart Taq ReadyMix, 2.5 μl of 25 mmol/l MgCl_2_ adjusted to 20 μl with water. *GAPDH* mRNA was used as normalizing transcript in reactions analogous to the above using 1 μl of 10 μmol/L *GAPDH* primer mix and 0.5 μl of 10 μmol/L *GAPDH* probe. For assay of interferon regulatory factor 1 (*IRF1*), *IRF1* mix (Hs00971959_m1), *GAPDH* mix (Hs99999905_m1) and TaqMan Universal PCR Master Mix (Part No. 4304437) were used (Applied Biosystems, Foster City, CA, USA) with PCR conditions as above. Real time PCR was run using an Eppendorf RealPlex (Hamburg, Germany) and gene relative expression was calculated using the ΔΔC_t_ method. Each sample was run in duplicate. Two standard calibrator mRNA isolates from brain were run on every plate when calibration between runs was necessary. Brain HIV RNA was quantified using cDNA derived from the brain total RNA isolated as above. The HIV gag/pol primer and probe sequences were from Palmer et al. (2003). The reaction contained 4 μl of cDNA, 12.5 μl of Sigma JumpStart Taq ReadyMix, 3.5 μl of 25 mmol/L MgCl_2_, 0.8 μl of 10 μmol/L HIV primer mix and 0.5 μl of 10 μmol/L HIV probe adjusted to 25 μl. PCR conditions were as above. RNA copy per microgram of total RNA was calculated using a standard curve using a previously quantified HIV-positive RNA sample as the primary standard. HIV RNA copy per gram of brain tissue was calculated using RNA extraction yield (mg/g) divided by the starting wet weight of the brain tissue sample.

### Statistics

HIV-infected subjects with and without HIV encephalitis (HIVE) were compared to HIV negatives and to each other using one-way analysis of variance with post hoc comparisons using Tukey’s test. The groups of HIV infected subjects with and without syndromic neurocognitive impairment were compared to each other and the HIV negatives as above. For correlation analysis in which there was little deviation from linearity such as for normalized T scores, Pearson’s product moment correlation was used. Spearman’s rank test was used when deviation from linearity was prominent, such as for IRF1. The significance of the correlations was corrected for the false discovery rate (Benjamini and Yekutieli, 2001) using alpha = 0.05. Using the HIV-infected subjects who underwent the substance abuse survey, multiple logistic regression models with stepwise elimination was used to determine whether nine categories of abuse in the PRISM survey were related to the two synaptic mRNAs. These models included any kind of substance abuse as one potential independent variable. The pooled students *t* test was used when two mutually exclusive groups were compared to each other (such as with major depression versus without, or with Hepatitis C infection versus without). When two correlations were compared to each other (such as the correlations between IRF1 and synaptic markers) the bootstrap approach was used (Efron 1979). The general significance level was *p* < 0.05 using a two tailed probability.

## Results

### PENK *and* DRD2L *expression*

**Fig. 1:**
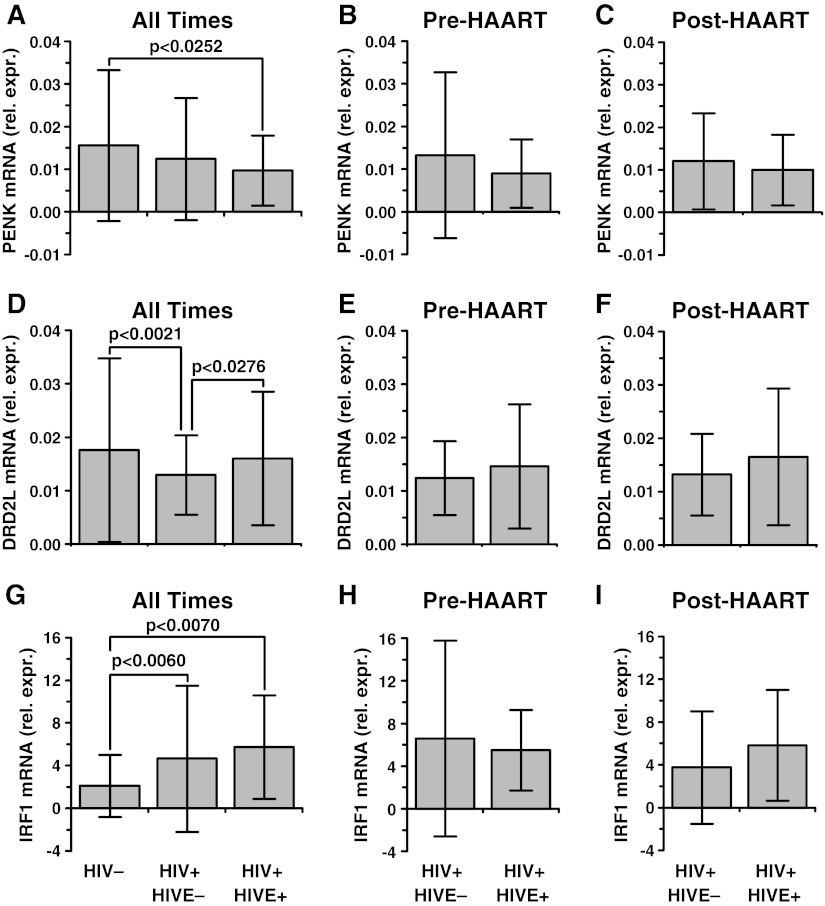
Preproenkephalin (*PENK*), dopamine receptor (*DRD2L*), and interferon response factor 1 (*IRF1*) mRNAs in frontal neocortex in 67 seronegative patients and 446 patients with HIV/AIDS (Panels **a**, **d**, **g**). “Rel. expr.” on the ordinate denotes mRNA expression relative to *GAPDH* mRNA. The 446 subjects with HIV/AIDS were subdivided into groups without and with HIV encephalitis (HIVE) (*n* = 354 and 92 respectively). Panels **b**, **c**, **e**, **f**, **h**, **i** show the composite data split into subjects who died before (*n* = 111 and 23) and during (*n* = 243 and 69) the era of highly active antiretroviral therapy (HAART). The between group analysis using one-way analysis of variance was significant for *PENK* (*F* = 3.45, *p* < 0.0335), *DRD2L* (*F* = 7.78, *p* < 0.0005), and *IRF1* (*F* = 7.03, *p* < 0.0010). *PENK* was lower in the subjects with HIVE (*p* < 0.0252); *DRD2L* was lower in the subjects without HIVE (*p* < 0.0021); *IRF1* was higher with and without HIVE (*p* < 0.0007 and 0.0060 respectively). The patterns were present prior to and during the era of HAART. Mean ± standard deviation

*PENK* had significant between-group effects comparing controls to HIV positives with and without HIVE (*F* = 3.45, *p* < 0.0335) (Fig. 1a). The subjects with HIVE had an average decrease of 37% (*p* < 0.0252, Tukey’s test); the subjects without HIVE did not have a significant decrease in *PENK* (*p* < 0.2049). The difference was evident both before and during the HAART era (Fig. 1b,c).

Gene array data have suggested that DLPFC *DRD2L* mRNA was regulated in HAND (Gelman et al. 2004) and this survey confirmed it (Fig. 1d). There was a significant between-group effect using one-way ANOVA *DRD2L* (*F* = 7.78, *p* < 0.0005). *DRD2L* was lower by an average of 26% in the HIV infected subjects *without* HIVE (*p* < 0.0021) but was not lower in the subjects *with* HIVE (*p* < 0.6117). The difference was evident both before and during the HAART era (Fig. 1e,f).

**Fig. 2:**
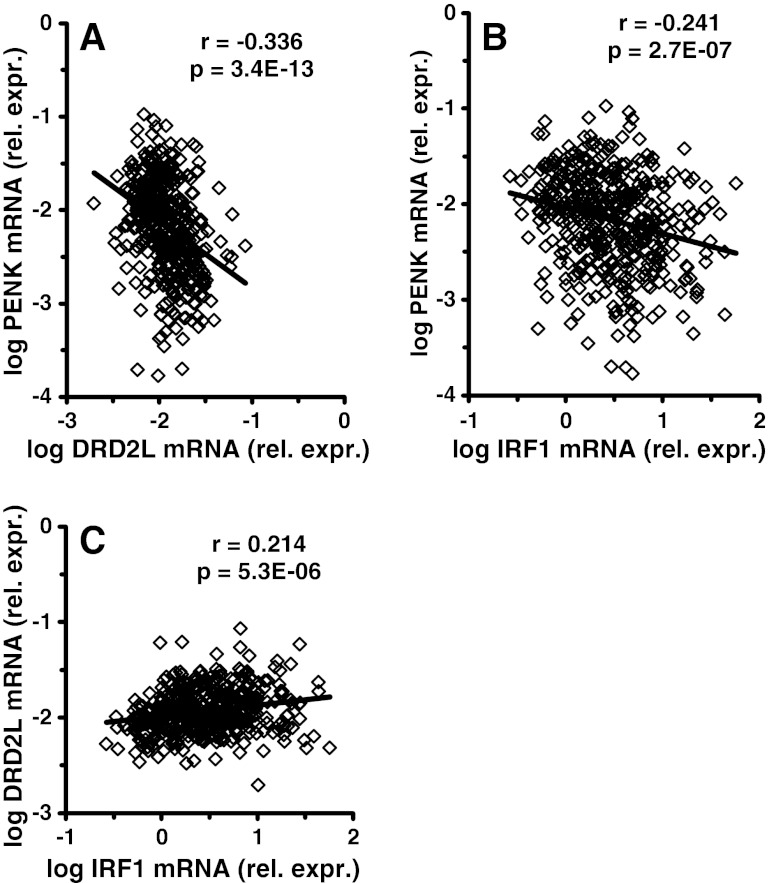
Changes in preproenkephalin (*PENK*) and dopamine receptor (*DRD2L*) mRNA expression in frontal neocortex in 446 HIV-infected subjects. *PENK* and *DRD2L* tended to run opposite to each other; the correlation coefficient was significant with a negative slope (**a**). When compared to the inflammatory marker *IRF1* mRNA, *PENK* and *DRD2L* both were significantly correlated. *PENK* was correlated with a negative slope (**b**); *DRD2L* was correlated with a positive slope (**c**). Using the bootstrap approach, the correlations in **b** and **c** are significantly different from each other (*p* < 0.001)

Results with or without HIVE suggested that *PENK* and *DRD2L* mRNA were not downregulated in the same subjects. Indeed, the two synaptic mRNAs appeared to run in opposite directions in HIV-infected subjects (Fig. 2a). The linear correlation coefficient between the two mRNAs after log transformation was negative and highly significant (*r* = −3.36, *p* = 3.34E-13).

### Relationship to interferon response

The above indicated that HIVE was related to the dysregulation of *PENK* and *DRD2L*. Brain specimens with HIVE usually have higher host neuroimmune responses such as interferon response gene (IFRG) expression (Masliah et al. 2004) and increased brain HIV RNA concentration (Achim et al. 1994). To determine whether IFRG expression was related to the synaptic mRNAs, a prototypal IFRG mRNA was assayed. Interferon regulatory factor mRNA (*IRF1*) (Gobin et al. 1999) was increased significantly (*F* = 7.03, *p* = 0.0010) by 2.2 fold in subjects without HIVE (*p* < 0.0060), and by 2.7 fold in the subjects with HIVE (*p* < 0.0007) (Fig. 1g). Correlation analysis showed that log *IRF1* was significantly and *negatively* correlated with *PENK* (*r* = −0.241, *p* < 2.70E-07, Pearson’s test) (Fig. 2b). Log *IRF1* was significantly and *positively* correlated with log *DRD2L* (*r* = +0.214, *p* < 5.30E-06) (Fig. 2c). Using the bootstrap approach the two correlations were significantly different from each other (*p* < 10E-06).

### Relationship to brain HIV RNA concentration

In contrast to the significant correlations with *IRF1*, correlation coefficients between the synaptic mRNAs versus log brain HIV RNA concentration were not significant. A weak threshold concentration of 10E + 05 copies of HIV RNA per gram of wet brain tissue was suggested that was significant for increased *DRD2L* (0.0131 ± 0.0081 below versus 0.0154 ± 0.0105 above, *p* < 0.019, student’s *t* test), but not for decreased *PENK* (*p* < 0.099).

### Relationship to neurocognitive impairment

**Fig. 3:**
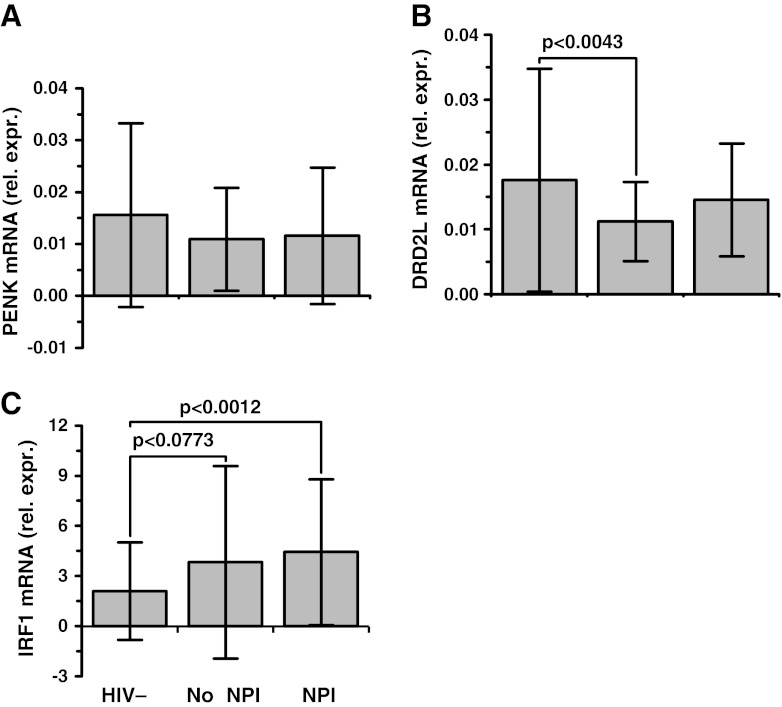
Preproenkephalin (*PENK*), dopamine receptor (*DRD2L*), and interferon response factor 1 (*IRF1*) mRNAs in frontal neocortex (**a**, **b** and **c** respectively). “Rel. expr.” on the ordinate denotes mRNA expression relative to *GAPDH* mRNA. On the abscissa 67 HIV negative subjects (HIV-) are compared to 146 HIV positive subjects (HIV+) with neurocognitive impairment (NPI), and 57 HIV+ subjects without NPI. The between group differences using one-way ANOVA were significant for *DRD2L* (*F* = 5.13, *p* < 0.0065) and *IRF1* (*F* = 6.38, *p* < 0.0020). *DRD2L* was significantly lower in subjects without NPI (*p* < 0.0043, Tukey’s test). Mean ± standard deviation

HIV infected subjects with and without neurocognitive impairment were compared to each other, and to seronegative controls (Fig. 3). One-way ANOVA showed a significant between group effect for *DRD2L* (*F* = 5.13, *p* = 0.0065) but not *PENK* (*F* = 2.36, *p* = 0.0962). The post hoc analysis showed that subjects who *did not have* impairment had a 36% decrease in *DRD2L* (*p* < 0.0043); the subjects who *did have* impairment had a decrease of 17% in *DRD2L* that was not statistically significant (*p* < 0.1619). We performed linear regression analysis using the composite neurocognitive impairment T score (a normalized measure of composite performance) and found a significant negative correlation with *DRD2L* (Table 2). The minus sign of the correlation coefficient indicated that lower *DRD2L* expression was linked with *stronger* performance (a higher T score) and a more favorable neurocognitive outcome overall.

**Table 2:** Correlation between normalized neurocognitive T scores and prefrontal synaptic mRNAs

Neurocognitive test domain	n ^a^	*DRD2L* mRNA ^b^		*PENK* mRNA	
		r ^c^	p ^d^	r	p
Verbal Fluency	215	−0.2450	0.0003*	0.0444	0.5168
Attention and working memory	211	−0.2180	0.0014*	0.0116	0.8661
Speed of Information processing	216	−0.1983	0.0034*	0.0232	0.7339
Motor	202	−0.1675	0.0171	0.0502	0.4778
Learning	218	−0.1653	0.0145	−0.0445	0.5127
Abstract Executive	208	−0.1461	0.0352	0.0363	0.6019
Memory	217	−0.1004	0.1404	−0.0916	0.1787
Composite T score, all tests	196	−0.2394	0.0007*	0.0051	0.9434

^a^n, number of subjects with available T score

^b^ *DRD2L* mRNA, dopamine receptor type 2 long splice isoform messenger RNA; *PENK* mRNA, preproenkephalin

^c^r, Pearson’s correlation

^d^p, Two-tailed probability

*Bold denotes significant statistically after controlling for the false discovery rate. The composite score does not require adjustment; its p-value is significant with or without correction

The composite diagnosis of neurocognitive impairment takes into account several component functional “domains” (Woods et al. 2004). To determine whether DLPFC *DRD2L* expression was correlated selectively with specific types of tasks, multiple correlations using the component domain T scores were computed (Table 2). Negative correlation coefficients were obtained for performance on tasks that required verbal fluency, working memory formation and rapid information processing. These r-values were significant after correction for the false discovery rate due to multiple comparisons. These tasks are driven characteristically by the frontal lobes, and are modulated by DLPFC dopaminergic tone (Parks et al. 1988; Sawaguchi et al. 1990; Goldman-Rakic 1996; Goldman-Rakic 1998; Gulledge and Jaffe 1998; Schlosser et al. 1998; Baddeley 2003; Tierney et al. 2008; Nagano-Saito et al. 2008; Kroener et al. 2009). For tasks driven primarily by temporal lobe circuits such as learning and memory formation (Squire and Zola-Morgan 1991), the correlation coefficients were not as strong and were not significant after adjustment for the false discovery rate (Table 2).

### Relationships to substance abuse history, depression, Hepatitis C virus and age

**Fig. 4:**
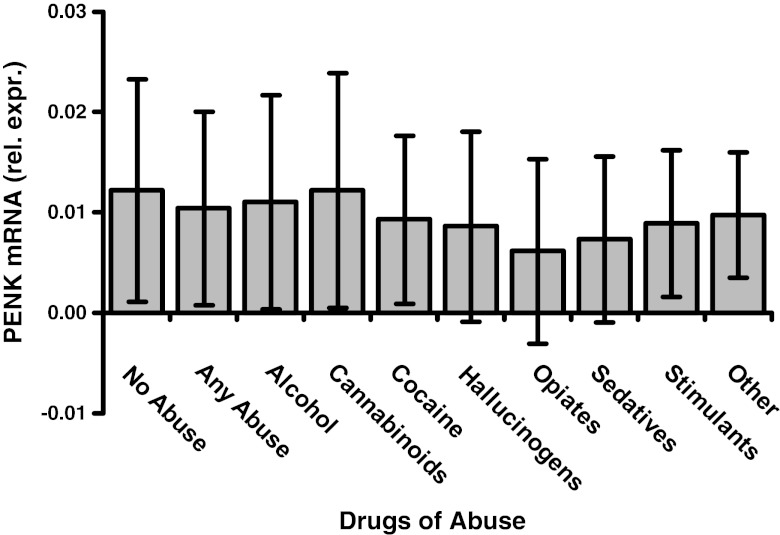
Preproenkephalin mRNA concentration (*PENK*) in frontal neocortex of HIV-infected subjects is plotted versus substance abuse categories. “Rel. expr.” on the ordinate denotes mRNA expression relative to *GAPDH* mRNA. Drug abuse was diagnosed using the Psychiatric Research Interview for Substance and Mental Disorders (PRISM). Drug abuse categories are given on the abscissa. “Other” denotes several substances such as inhalants. See “Results” for the number of subjects in each category. *PENK* was not significantly different in any substance abuse category using multiple regression models. Compared to subjects without any abuse (left bar), eleven subjects with opiate abuse had a trend towards having significantly less *PENK* using stepwise regression models that was of marginal significance (see “Results”). *DRD2L* mRNA also showed no significant group effect (not illustrated). Mean ± standard deviation

Substance use survey results were available in 194 of the HIV infected subjects. 76 had a least one substance of abuse and 118 had no abuse. The number of subjects in each category of abuse was: alcohol = 50; cannabinoids = 28; cocaine = 34; hallucinogens = 8; opiates = 11; sedatives =17; stimulants = 28; other = 7 (Fig. 4). Multiple regression models were used to address the fact that multiple substances may be abused. Using DLPFC *PENK* as the response, the models showed that all p-values were greater than 0.2, and the overall p-value was not significant (*F* = 1.293, *p* = 0.2505) using the Bayesian Information Criterion (BIC). A potentially important p-value for 11 subjects with opiate abuse of 0.0141 was obtained using Akeike’s Information Criterion (AIC), implying a possible relationship between opiate abuse and lower *PENK* expression. Multiple regression models using *DRD2L* as the response showed that all of the p-values for the individual categories of abuse were above 0.15, and the overall p-value was not significant (*F* = 1.03, *p* = 0.418).

The high prevalence of major depressive disorders (MDD) in HIV-infected cohorts (Berger-Greenstein et al. 2007) could influence opioidergic and dopaminergic systems (Peckys and Hurd 2001; Tremblay et al. 2005; Kennedy et al. 2006) and/or the neurocognitive output of the DLPFC (Ottowitz et al. 2002; van der Werf-Eldering et al. 2010). In 91 subjects with MDD versus 100 without it, neither *PENK* (0.0105 ± 0.0087 vs 0.0123 ± 0.0120, *p* < 0.264) nor *DRD2L* (0.0130 ± 0.0095 vs 0.0132 ± 0.0079, *p* < 0.892) was significantly different.

Hepatitis C virus (HCV) infection can influence HAND, especially in the presence of MDD (Clifford et al. 2005). The two synaptic mRNAs were not expressed differently comparing subjects with (n = 79) and without (n = 116) documented HCV infection (*PENK*: 0.0107 ± 0.0102 vs 0.0117 ± 0.0104, *p* < 0.504; *DRD2L*: 0.0139 ± 0.0079 vs 0.0147 ± 0.114, *p* < 0.586).

Advanced age has been reported to be associated with lower DRD2 binding availability to the extent of about 0.6% per year (Antonini et al. 1993; Volkow et al. 2000), and also with higher brain inflammation (Dickson et al. 1992). The HIV infected subjects in the autopsy cohort were slightly younger than controls (4 years on average, see Table 1), and raised the possibility that a younger average age might have influenced neurochemical outcomes. The *t*-test of the coefficient in a regression model testing the effects of HIV and age together showed that *IRF1* was higher (*p* < 0.001), *PENK* was lower (*p* = 0.022) and *DRD2L* was lower (*p* = 0.0597). Thus, age did not drive the changes observed in HIV infected subjects substantially, although a small influence of age on these outcomes remains difficult to exclude completely.

## Discussion

The study confirms that mRNA changes associated with altered synaptic transmission in frontal neocortex are prevalent in people with HIV/AIDS. At least 40% of the HIV infected population had downregulation of at least one of the two synaptic mRNAs. The abnormal synaptic mRNA pertaining to dopaminergic neural transmission (*DRD2L*) was correlated significantly with neurocognitive impairment, while the abnormal enkephalin-related mRNA (*PENK*) was not correlated with impairment independently. The fact that the two synaptic mRNAs were regulated in the opposite direction from each other implies that the changes do not reflect a generalized loss of synapses in the setting of neurodegeneration. Instead, the functional implications of the results pertain to synaptic plasticity and the circuit level output of the frontal lobes. Neurotransmitter “tone” is dynamically regulated in many ways. Modulating the concentration of the neurotransmitter presynaptically (illustrated with *PENK* regulation) or changing the number of receptors postsynaptically (illustrated with *DRD2L* regulation) represent established aspects of synaptic plasticity (Malenka and Nicoll 1993; Elgersma and Silva 1999; Citri and Malenka 2008). Changes in synaptic tone at the cellular level produce shifts in neuronal excitation; dopaminergic and opioidergic tone both modify neuronal excitability at the cellular level (Xie and Lewis 1995; Otani et al. 2003). In turn, synaptic plasticity produces coordinated changes in the output of neural networks (Citri and Malenka 2008; Boulanger 2004). Clinically, circuit-level changes in DLPFC output modify neurocognitive test performance on tasks pertaining to verbal fluency, working memory formation, and speed of processing (Sawaguchi et al. 1990; Gulledge and Jaffe 1998; Schlosser et al. 1998; Goldman-Rakic 1996; Tierney et al. 2008). All of those kinds of tasks were correlated significantly with DLPFC *DRD2L* expression in HIV/AIDS (Table 2). Dopamine receptive neurons in the DLPFC that express *DRD2L* play a critical role in controlling the output of frontal lobe circuits (Goldman-Rakic 1998; Wang et al. 2004a, b; Nagano-Saito et al. 2008; Kroener et al. 2009). In primates these neurons are concentrated in the excitatory output neurons in cortical Lamina V. Their excitation state fundamentally controls DLPFC circuit output (Lidow et al. 1998; Wang et al. 2004a, b; Cohen et al. 2002). Enkephalinergic neurons and cognate delta opioid receptors in DLPFC also are localized in Lamina V and vertically connected neurons in Laminae II and III (McGinty et al. 1984; Mansour et al. 1993). The cortical enkephalinergic system also influences frontal lobe output (Verdejo et al. 2005). Together these abnormal synaptic systems can exert substantial control on the functional output of the frontal lobe circuitry, and thus, might drive frontal lobe dysfunction in HAND and HIVE (Woods et al. 2004).

**Fig. 5:**
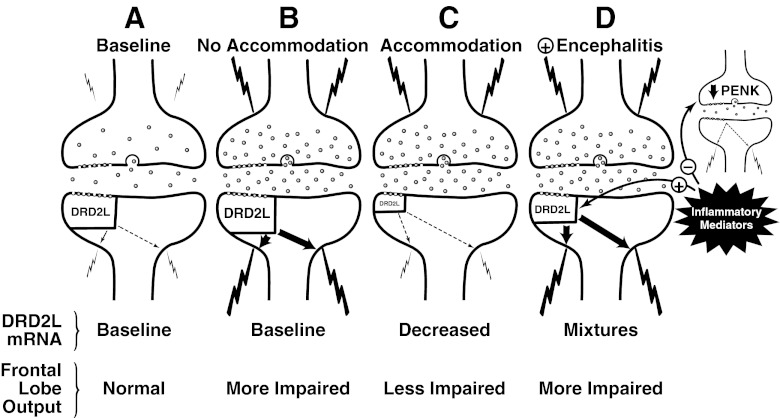
Prefrontal dopamine receptive neurons in HIV infected subjects often have abnormal mRNA expression of genes that are critical for synaptic transmission. The proposed functional implications for a dopaminergic synapse of a Lamina V pyramidal neuron are illustrated in four scenarios designated **a**, **b**, **c** and **d**. Scenario A depicts baseline dopaminergic tone in a normal person. Scenario B depicts no change in postsynaptic *DRD2L*; subjects fitting that picture were more likely to have neurocognitive impairment and high excitatory frontal lobe output. Scenario C depicts a decrease in postsynaptic *DRD2L* due to accommodation; subjects fitting that picture were less likely to have neurocognitive impairment and lower excitatory output. Scenario D depicts HIV encephalitis which produces an increase in inflammatory mediators; subjects fitting that picture probably had bidirectional changes in *DRD2L*, including increased expression when inflammation and HIVE were present. Inflammation and HIVE also were associated with decreased *PENK* expression, which ran opposite to *DRD2L* regulation. The nature of the suggested connection between dopaminergic and enkephalinergic transmission remains to be elucidated. Key: The presynaptic bouton is on top and postsynaptic bouton is below. For the purpose of illustration, the postulated driver of postsynaptic *DRD2L* expression is increased presynaptic dopaminergic tone (the cause is not clear). A larger size of the *DRD2L* symbol denotes higher receptor mRNA expression. Bolder arrows denote stronger receptor transduction. Bolder lightning bolts denote stronger depolarization or high firing rates. A high density of presynaptic vesicles symbolizes higher presynaptic tone

The type 2 dopamine receptor (*DRD2L*) is a pivotal point of neural circuits that originate in the midbrain, such as the mesofrontal circuit in DLPFC (Goldman-Rakic 1998) and the classical “direct” striatopallidal circuit (Gerfen et al. 1990). *DRD2L* expression often is a transcriptionally active marker gene in dopamine receptive neurons that responds dynamically to changes in synaptic dopaminergic tone (Gerfen et al. 1990; Angulo et al. 1991; Volkow et al. 1999; Trantham-Davidson et al. 2004; Seamans and Yang 2004; Sesack and Carr 2002), and also can modulate *PENK* expression (Gerfen et al. 1991; Morris and Hunt 1991; Przewłocka and Lasoń 1995; Steiner and Gerfen 1999). Regulating dopaminergic tone in the mesofrontal circuitry has consequences that are potentially both favorable and unfavorable. DLPFC output responds to dopaminergic tone in an “inverted U” type of dose–response curve, in which excessive deviation in either direction modifies synaptic tone and can produce DLPFC dysfunction (Nagano-Saito et al. 2008; Cools and D’Esposito 2011; Monte-Silva et al. 2009). One stimulus that shifts the inverted U response curve and drives DLPFC *DRD2L* expression lower is increased presynaptic dopaminergic tone (Ito et al. 2011; Sesack and Carr 2002; Volkow et al. 1999). In HIV/AIDS the functional output of the DLPFC was most favorable when *DRD2L* expression was downregulated, which implies a compensatory shift to a more favorable point on the inverted U response curve. Conversely, DLPFC output was less favorable when *DRD2L* showed no net downregulation (Fig. 3), which implies a less optimal point on the inverted U response curve. Thus, decreased *DRD2L* expression probably reflects a compensatory lowering of dopaminergic set point to synaptically accommodate, and stabilize DLPFC output (Floresco and Magyar 2006). We suggest that this is the most likely reason why failing to drive dopaminergic tone downward was associated with less beneficial outcomes. The fact that an unfavorable neurocognitive outcome was associated with “normal” *DRD2L* expression is seemingly paradoxical. But those results are in substantial accord with clinical observations, including some work with HIV-infected subjects. Using functional magnetic resonance spectroscopy (MRS) it was shown that many subjects with task-driven functional abnormalities of frontostriatal circuitry, and/or glial activation (Melrose et al. 2008; Ernst et al. 2002; Meyerhoff et al. 1999), *do not have* HIV-associated neurocognitive impairment. Conversely, many subjects without those synaptic accommodations (i.e. appear “normal” in the brain images) *do have* impairment. These types of clinical observation in HIV/AIDS, in conjunction with the vast experimental literature on prefrontal dopaminergic systems, suggest the scenarios that are illustrated in Fig. 5: 1) DLPFC dopaminergic circuit accommodation does occur in some subjects with HIV/AIDS. 2) When it occurs it appears to be beneficial to frontal lobe output as depicted in scenario C. 3) When it fails to occur it is potentially unfavorable as depicted in scenario B. 4) Brain inflammation and/or HIVE (represented by the interferon response gene *IRF1*) seems to drive *DRD2L* expression upward and has an unfavorable influence on DLPFC dopaminergic tone, and possibly, enkephalinergic tone as in Scenario D.

Brain imaging in subjects with HAND has suggested that neostriatal ligand binding availability is low for postsynaptic DRD2 and the presynaptic dopamine reuptake transporter (DAT). Those results are generally taken to suggest that striatal dopaminergic tone is abnormal, although the technical approaches used were not sufficiently sensitive to detect a change in the DLPFC (Chang et al. 2008). Indeed, assay of synaptic mRNAs was the chosen approach at autopsy primarily because detection of neocortical protein elements was out-of-range. Beyond the problem of detection, each dopaminergic circuit has interdependent regulatory properties that often run opposite to each other (Wilkinson 1997; Kellendonk et al. 2006). Despite the technical differences, autopsy results agree that dopaminergic transmission is related to HIV-associated neurocognitive dysfunction. The decreased transcription regulation of *DRD2L* at autopsy is consistent with a loss of raclopride binding availability of DRD2 in the neostriatum (Chang et al. 2008). As well, the suggestion that increased presynaptic tone could drive the changes (as illustrated in Fig. 5) is consistent with having decreased dopamine binding availability of DAT. In the latter scenario, a higher concentration of endogenously synthesized dopamine in the synaptic cleft could compete with exogenous radiotracer dopamine and reduce its binding availability, which is what was observed in neostriatum (Chang et al. 2008).

The mechanism that produces altered dopaminergic transmission in HIV/AIDS is of great interest and needs to be elucidated. Experimental evidence shows that HIV-1 can interact directly with dopaminergic synapses. Specifically, the concentration of HIV-1 transactivator of transcription protein (Tat) which is known to be toxic, influences the physiochemical interplay between dopamine and neostriatal DAT, and could disturb dopamine binding availability to DAT (Zhu et al. 2011). Pertinent to that suggestion, the autopsy survey showed that the concentration of HIV gag/pol RNA in the brain was not correlated significantly with *DRD2L* expression. A history of taking HAART to suppress HIV replication also showed no apparent relationship at autopsy. Those observations imply that the concentration of HIV-1 proteins such as Tat do not directly drive *DRD2L* regulation. The same could apply to DAT binding availability, although the issue was not addressed specifically at autopsy because presynaptic DAT and its mRNA could not be detected consistently in the DLPFC.

One implication for future work is that inflammatory type mediators such as the IFNs might be directly involved with dopaminergic transmission in HIV/AIDS. Autopsy data implied that inflammation was associated with higher *DRD2L* and lower *PENK* (Fig. 2). This was illustrated using a prototypal IFN-γ-responsive gene (IFRG); other inflammatory mediators produced equivalent results (not illustrated). IFNs and IFRGs are neuroimmune mediators of prime interest because they are strongly expressed in virus infected brain tissue including HIVE (Masliah et al. 2004; Sas et al. 2009). The connection between the IFRG expression and synapse biology in HIV/AIDS agrees fundamentally with a broad base of experimental data (see the extensive list of pertinent citations in the introduction). IFN and other neuroimmune mediators modify synaptic functions directly by driving IFN receptors on neurons (Neumann et al. 1997; Delhaye et al., 2006; Mizuno et al. 2008; Sas et al. 2009; Dafny 1998), and indirectly by increasing the synthesis of IFRGs. IFNs act upon two types of receptors that drive the expression of hundreds of IFRGs (Boehm et al. 1997; Theofilopoulos et al. 2005). Examples of indirect action of IRF-1 include the induction of class I major histocompatibility antigens, and nitric oxide synthesis (Martin et al. 1994; Hobart et al. 1997), which in turn modify synaptic long term potentiation (Garthwaite and Boulton 1995; Goddard et al. 2007). Experimental data show that increased IFN-γ also lowers *PENK* mRNA expression (Low et al. 1992; Negro et al. 1992; Ovadia et al. 1996), as observed in the subjects with HIVE (Fig. 1). Experimentally, the proposed linkage between heightened immune responses and neural transmission can operate in both directions via feedback loops. For example, opioid neuropeptides including *PENK* drive neuroimmune cellular responses forward, including increased macrophage and lymphocyte infectivity with HIV-1 (Nath et al. 2002; Wetzel et al. 2000). A feedback loop is suggested in which heightened neuroimmunity leads to *PENK* downregulation and weakens HIV-1 replication. Dopamine and serotonin also modify macrophage HIV-1 replication via their cognate receptors, and could engage in similar feedback loops (Maneglier et al. 2008; Gaskill et al. 2009). The suggested scenarios need to be confirmed in model systems focusing on receptor proteins and their endogenous agonists.

Substance abuse is prevalent in HIV-infected populations. It is associated with altered neural transmission (Wolf 2002; De Vries and Shippenberg 2002), including lower striatal *DRD2* mRNA expression, and especially with cocaine and methamphetamine use (Volkow et al. 1999; Goldstein and Volkow 2002; Chang et al. 2008). Substance abuse also can change neurocognitive function pertaining to DLPFC output (Nath et al. 2002). The linkage of substance abuse with lower DRD2 expression implies that it also could be associated with synaptic “accommodation” as illustrated in Fig. 5, which in turn, could prove to be *favorable* in terms of neuropsychological performance. We did not detect associations between substance use and neurochemical changes at autopsy using a self report survey, probably because a postmortem survey is affected by multiple confounding factors during a human lifetime. Drugs are often abused intermittently, using complex combinations of substances, for varying durations. Acute intoxication, binging, fatal overdoses and undocumented abstinence periods all can influence synaptic tone, yet cannot be controlled for generally in an autopsy survey. Another potentially important influence to consider is nicotine dependence, which can stimulate dopamine release in the striatal reward pathway and produce loss of radiotracer ligand binding to *DRD2*. Nicotine use modified the strength of the association between HIV infection and striatal DAT binding availability in the study by Chang et al. (2008). Smoking histories were not available in the autopsy survey, so the lower *DRD2L* expression with HIV/AIDS might have been influenced by nicotine use. The great complexity introduced by multiple historical variables suggests that the influence of substance abuse needs to be unraveled using corollary experimental paradigms of HIV infection.

In addition to acquired factors that occur intermittently before death, innate factors such as host genetic polymorphism might be important. The host genome could produce enduring effects on synaptic mRNA regulation primarily because it does not fluctuate with time. *DRD2L* gene polymorphism modifies *DRD2L* mRNA expression and changes the functional output of the DLPFC (Duan et al. 2003; Zhang et al. 2007). As well, enzymatic degradation of synaptic dopamine by catechol-O-methyltransferase (COMT) is influenced by host polymorphism of *COMT* which in turn, can modify dopaminergic output of the DLPFC (Egan et al. 2001). In that scenario a subject would acquire low *DRD2L* or *PENK* expression (and potential synaptic accommodation) prior to acquiring HIV/AIDS. If host genes can indeed drive *DRD2L* expression, vulnerable subjects with inherently less favorable genes, and weaker synaptic accommodation, could be identified a priori and treated aggressively with measures to stimulate accommodation and promote more favorable neuropsychological outcomes. Similarly, less vulnerable subjects might be identified clinically. Neuropharmacological manipulation of opioidergic and dopaminergic tone represents a logical way to mimic naturally occurring synaptic accommodation to improve neurocognitive function (Hille et al. 2001; Cohen and Servan-Schreiber 1992). The suggested approach acts downstream from brain neuroimmune activation, and is an attractive site of action because neural immunity must be preserved in order to control brain HIV-1 replication, suppress viral latency or participate in therapeutic virus eradication paradigms.

### Summary

Neurochemical measurements in 512 autopsy brain specimens lend strong credence to the hypothesis that synaptic modulation occurs often in the DLPFC of subjects with HIV/AIDS. Dopaminergic and opioidergic neurotransmitter systems were regulated transcriptionally and exhibited various linkages to brain neuroimmunity, brain pathology and brain function. The most important association that was established is that decreased expression of *DRD2L* in frontal neocortex was linked to a more favorable neuropsychological outcome. Coordinated neuropharmacological manipulation of such neurotransmitter imbalances could modulate the brain circuits that drive neurocognitive impairment or provide synaptic accommodation in HIV/AIDS. Protecting circuit level function pharmacodynamically is a therapeutic approach that preserves host neural immunity and is potentially complementary to HAART.
